# Advantages of McKeown minimally invasive oesophagectomy for the treatment of oesophageal cancer: propensity score matching analysis of 169 cases

**DOI:** 10.1186/s12957-022-02527-z

**Published:** 2022-02-25

**Authors:** Jun Xie, Lei Zhang, Zhen Liu, Chun-lei Lu, Guang-hui Xu, Man Guo, Xiao Lian, Jin-Qiang Liu, Hong-Wei Zhang, Shi-ying Zheng

**Affiliations:** 1 Department of Thoracic Surgery, The First Affiliated Hospital of Soochow University, Shizi Street No. 188, Suzhou, 215006 Jiangsu China; 2 The Key Laboratory of Biomedical Information Engineering of Ministry of Education, School of Life Science and Technology, Xi’an Jiaotong University, Xi’an, Shanxi Province China; 3 Department of General Surgery, Peking Union Medical College Hospital, Chinese Academy of Medical Sciences & Peking Union Medical College, Beijing, 100730 China; 4 Digestive Diseases Center of Wuxi Mingci Hospital, No. 599 Zhongnan Road, Jinxing Street, Wuxi City, 214000 Jiangsu Province China; 5 Department of General Surgery, Xijing Hospital of Digestive Diseases, The Fourth Military Medical University, Xi’an, 710033 Shan Xi Province China

**Keywords:** Oesophageal cancer, McKeown MIE, Open oesophagectomy, Prognosis

## Abstract

**Background:**

Oesophagectomy, the gold standard for oesophageal cancer treatment, causes significantly high morbidity and mortality. McKeown minimally invasive oesophagectomy (MIE) is preferred for treating oesophageal malignancies; however, limited studies with large sample sizes focusing on the surgical and oncological outcomes of this procedure have been reported. We aimed to compare the clinical safety and efficacy of McKeown MIE with those of open oesophagectomy (OE).

**Patients and methods:**

Overall, 338 oesophageal cancer patients matched by gender, age, location, size, and T and N stages (McKeown MIE: 169 vs OE: 169) were analysed. The clinicopathologic features, operational factors, postoperative complications, and prognoses were compared between the groups.

**Results:**

McKeown MIE resulted in less bleeding (200 mL vs 300 mL, *p*<0.01), longer operation time (335.0 h vs 240.0 h, *p*<0.01), and higher number of harvested lymph nodes (22 vs 9, *p*<0.01) than OE did. Although the rate of recurrent laryngeal nerve injury in the two groups was not significantly different, incidence of anastomotic leakage (8 vs 24, *p*=0.003) was significantly lower in the McKeown MIE group. In addition, patients who underwent McKeown MIE had higher 5-year overall survival than those who underwent OE (69.9% vs 40.4%, *p*<0.001).

**Conclusion:**

McKeown MIE is proved to be feasible and safe to achieve better surgical and oncological outcomes for oesophageal cancer compared with OE.

## Background

Oesophageal cancer (EC) is the sixth-leading cause of cancer-related death globally [1] and China is among the countries facing the highest risk of EC [2]. The mortality of EC in China is the highest in the world, with an incidence of 16.7 per 100,000 person-years and a death rate of 13.4 per 100,000 person-years [3]. For resectable EC, oesophagectomy combined with chemoradiotherapy remains the mainstay of multimodality treatment [4–8].

Open oesophagectomy (OE) is correlated with remarkably high rates of morbidity and mortality during the perioperative period and is considered as one of the most traumatic and extensive surgeries in cancer [9]. Therefore, with the development of minimally invasive technique [10, 11], McKeown minimally invasive oesophagectomy (MIE), which was first reported in 2000 and achieved similar or better clinical outcomes compared with OE [12], has become increasingly popular and is favourably performed at most academic centres. To date, several studies that compared the outcomes of OE and MIE have been published. Most of them reported that MIE reduced the surgical access-related trauma, which resulted in shorter hospitalisation and lower rates of respiratory complications and wound infections [13–17].

However, the surgical and oncological outcomes of MIE remain controversial. Nonrandomised studies have shown patients treated with MIE have lower rates of major complications than those treated with OE [16, 18, 19]. Only one RCT enrolling 115 patients from five centres in the Netherlands reported that MIE was associated with a low rate of pulmonary infection, but it lacked the power to detect any oncologic difference [20]. In addition, a large systematic review concluded that the actual benefits of MIE over OE remain unclear in terms of short and long-term outcomes [21]. The cervical anastomosis performed in McKeown MIE is an invasive procedure associated with a high risk of injury in the recurrent laryngeal nerve (RLN) and anastomotic leakage [22], although cervical fistula remains a manageable complication in case of leakage [23]. In addition, none of the studies above used a median follow-up time > 40 months [15, 24–29].

Therefore, the current study aimed to compare the surgical and oncological outcomes in patients with EC who underwent OE or McKeown MIE performed in two independent centres, respectively.

## Patients and methods

This retrospective study has been approved by the Xijing Hospital Ethics Committee in compliance with the ethical principles stated by the Declaration of Helsinki. From August 2010 to December 2014, 463 EC patients who underwent McKeown MIE in Xijing Hospital and 169 EC patients who underwent OE in the First Affiliated Hospital of Soochow University were retrospectively included.

The eligibility criteria were as follows: (1) diagnosis confirmed by oesophagoscopy and pathology of the biopsies; (2) underwent oesophagectomy (McKeown MIE or OE); (3) without pre-operative neoadjuvant chemotherapy and/or radiotherapy; (4) clinical T1-3N0-1M0 stage; (5) respiratory function tolerability under double-lung ventilation for thoracotomy operation; and (6) no previous thoracic, hiatal, or bariatric surgery. Exclusion criteria were as follows: (1) underwent palliative resection and (2) incomplete records. Surgeons in both centres were experienced in OE, and those performing McKeown MIE were required to have done at least 40 McKeown MIEs owing to the learning curve of the procedure.

The 463 patients who underwent McKeown MIE were matched with 169 patients who underwent OE using a 1:1 propensity score matching (PSM) by gender, age, tumour location and size, and T and N stage. After the matching, a total of 338 EC patients (McKeown MIE: 169 vs OE: 169) were included.

The 169 patients in the McKeown MIE group received cervical anastomosis and omentoplasty while patients in the OE group received intrathoracic anastomosis. The gastric tube for substitution was used for all the patients in the McKeown MIE group and the entire stomach for substitution was performed in the OE group.

### Postoperative follow-up

Patients were followed up from the first month when discharged, and every three months in the following two years, then every 6 months by outpatient service via telephone as per National Comprehensive Cancer Network guidelines [30].

### Data collection

Preoperative data including gender, age, preoperative smoking history, tumour site, preoperative diet, and comorbidities were prospectively collected. Blood loss and operative time were recorded as intraoperative data. Postoperative data included histologic type, tumour size, tumour invasion, lymph node metastasis, harvested lymph nodes, postoperative hospitalisation, R0 resections, reoperation, length of intensive care unit stay, complications, and in-hospital/30-day death. The TNM stage was defined according to the 7th edition of the AJCC Staging Manual.

### Statistical analyses

The abnormally distributed continuous variables were expressed as median value (interquartile range) and categorical variables were expressed as numbers (percentages). PSM was performed using the R software (3.6.1) with Package MatchIt (version 4.1.0) to adjust for confounding variables including sex, age, location, size, and T and N stage between patients who underwent McKeown MIE and OE [31]. The abnormally distributed continuous variables were compared using Mann–Whitney *U* test. The categorical variables were compared using *χ*^2^ test or Fisher’s exact test. Survival curves were drawn using Kaplan–Meier methods and compared using the log-rank test. A two-tailed *p* value less than 0.05 was considered statistically significant. The data were analysed using the SPSS software (version 16.0, Chicago, IL).

## Results

From August 2010 to December 2014, 632 EC patients received oesophagectomy at two independent centres. Of them, 463 underwent McKeown MIE and 169 underwent OE. After PSM by gender, age, location, size, and T and N stage, a total of 338 EC patients with matched pairs (McKeown MIE: 169 versus OE: 169) were included. Clinical and pathological features of the enrolled patients are summarised in Table 1.

**Table 1:** Clinical characteristics of the patients with oesophageal cancer

Clinical characteristics	MIE group (*n*=169)	OE group (*n*=169)	*P*
Sex			0.804
Male	126 (74.6%)	124 (73.4%)	
Female	43 (25.4%)	45 (26.6%)	
Age			0.229
≤ 49	16 (9.5%)	13 (7.7%)	
50–59	46 (27.2%)	47 (27.8%)	
60–69	89 (52.7%)	78 (46.2%)	
70–79	18 (10.7%)	29 (17.2%)	
≥ 80	0 (0.0%)	2 (1.2%)	
Preoperative smoker			0.446
Yes	79 (46.7%)	86 (50.9%)	
No	90 (53.3%)	83 (49.1%)	
Tumour location (%)			0.967
Upper	16 (9.5%)	17 (10.1%)	
Mid	90 (53.3%)	91 (53.8%)	
Lower	63 (37.3%)	61 (36.1%)	
Preoperative diet			0.109
Solid	24 (14.2%)	20 (11.8%)	
Semi-liquid	84 (49.7%)	96 (56.8%)	
Total liquid	36 (21.3%)	41 (24.3%)	
Water	25 (14.8%)	12 (7.1%)	
Comorbidity	37 (21.9%)	28 (16.6%)	0.214
Hypertension	16 (9.5%)	11 (6.5%)	0.316
Diabetes	6 (3.6%)	7 (4.1%)	0.777
Coronary artery disease	5 (3.0%)	4 (2.4%)	1.000
COPD	7 (4.1%)	4 (2.4%)	0.358
Arrhythmia	2 (1.2%)	2 (1.2%)	0.174
Liver cirrhosis	1 (0.6%)	0 (0.0%)	1.000

*COPD* chronic obstructive pulmonary disease

EC was diagnosed and confirmed by two experienced pathologists. Of the 169 cases, 162 cases in the McKeown MIE group and 148 in the OE group were squamous cell carcinoma. The remaining were cases of adenocarcinoma (3), adenosquamous carcinoma (3), and small cell carcinomas (1) in the McKeown MIE group and adenocarcinomas (10), adenosquamous carcinomas (5), neuroendocrine carcinoma (2), small cell carcinoma (2), and sarcomas (2) in the OE group. Postoperative histologic features and tumour size had no significant difference between patients in the two groups (Table 2).

**Table 2:** Surgical and postoperative pathologic information of the patients with oesophageal cancer

Clinical variables	MIE group (*n*= 169)	OE group (*n*=169)	*P*
Substitution type			<0.01
Entire stomach	0	169 (100%)	
Gastric tube	169 (100%)	0	
Anastomotic type			<0.01
Cervical anastomosis	169 (100%)	0	
Intrathoracic anastomosis	0	169 (100%)	
Omentoplasty			<0.01
Yes	169 (100%)	0	
No	0	169 (100%)	
Histologic type			0.100
Squamous cell carcinoma	162 (95.9%)	148 (87.6%)	
Adenocarcinoma	3 (1.8%)	10 (5.9%)	
Adenosquamous carcinoma	3 (1.8%)	5 (3.0%)	
Neuroendocrine carcinoma	0 (0.0%)	2 (1.2%)	
Small cell carcinoma	1 (0.6%)	2 (1.2%)	
Sarcoma	0 (0.0%)	2 (1.2%)	
Tumour size (mean ± SD, cm)	4.78 ± 2.17	4.80 **±** 2.05	0.879
T stage			0.314
T1	21 (12.4%)	20 (11.8%)	
T2	47 (27.8%)	49 (29.0%)	
T3	100 (59.2%)	82 (48.5%)	
T4	1 (0.6%)	18 (10.7%)	
N stage			0.974
N0	105 (62.1%)	104 (61.5%)	
N1	53 (31.4%)	54 (32.0%)	
N2	7 (4.1%)	8 (4.7%)	
N3	4 (2.4%)	3 (1.8%)	

McKeown MIE was related with less bleeding (200 mL vs 300 mL, *p*<0.01), more harvested lymph nodes (22 vs 9, *p*<0.01) but longer operation time (335.0 h vs 240.0 h, *p*<0.01) than those of OE (Table 3). In addition, no difference was found regarding the rate of metastatic lymph nodes as well as the number of reoperations between the two groups. All of the patients underwent R0 resection (Table 3).

**Table 3:** Surgical outcomes of the patients with oesophageal cancer

Clinical variables	MIE group (*n*=169)	OE group (*n*=169)	*P*
Blood loss (median, IQR, ml)	200.0 (150.0, 300.0)	300.0 (250.0, 450.0)	<0.01
Operative time (median, IQR, min)	335.0 (280.0, 385.0)	240.0 (185.0,285.0)	<0.01
Lymph nodes harvested			
Median (IQR)	22 (17, 30)	9 (6, 15)	<0.01
Mean ± SD	24.5 ± 10.0	11.2 ± 7.9	<0.01
Number of metastasis nodes			
Median (IQR)	0 (0, 1.0)	0 (0, 1.0)	0.730
Mean ± SD	1.2 ± 3.2	0.98 ± 2.0	0.406
Rate of metastasis nodes	64 (37.9%)	67 (40.9%)	0.577
R0 resections	169 (100%)	169 (100%)	1.000
Reoperation	2 (50.0%)	2 (50.0%)	1.000

As shown in Table 4 for the postoperative complications, there were no intra-operative death cases in either group. McKeown MIE was significantly associated with lower rates of minor complications and major complications compared with OE (minor: 13.0% vs 22.5%, *p*=0.023; major: 17.8% vs 27.8%, *p*=0.028). Less patients who underwent McKeown MIE experienced pneumonia (5 vs 17, *p*=0.008) and anastomotic leakage (8 vs 24, *p*=0.003) postoperatively than those who underwent OE. Patients in the McKeown MIE group also had shorter hospitalisation (10 days vs 12 days, *p*<0.01) and a trend of less in-hospital/30-day mortality (2 vs 8, *p*=0.054). Other complications showed no significant difference between the two groups (*p*>0.05, Table 4).

**Table 4:** Complications of the patients with oesophageal cancer

Complications	MIE group (*n* =169)	OE group (*n* = 169)	*P*
Minor complication	22 (13.0%)	38 (22.5%)	0.023
Pneumothorax	5 (3.0%)	8 (4.7%)	0.396
Atelectasis	6 (3.6%)	5 (3.0%)	0.759
Pneumonia	5 (3.0%)	17 (10.1%)	0.008
Arrhythmia	4 (2.4%)	3 (1.8%)	1.000
Wound infection	2 (1.2%)	5 (3.0%)	0.448
Major complication	30 (17.8%)	47 (27.8%)	0.028
Pneumonia	2 (1.2%)	6 (3.6%)	0.283
RLN injury	4 (2.4%)	4 (2.4%)	0.638
Chylothorax	3 (1.8%)	2 (1.2%)	1.000
Anastomotic leakage	8 (4.7%)	24 (14.2%)	0.003
Delayed gastric emptying	4 (2.4%)	3 (1.8%)	1.000
Tracheo-bronchial injury	0 (0.0%)	3 (1.8%)	0.248
Anastomotic stricture	7 (4.1%)	3 (1.8%)	0.199
Respiratory failure	2 (1.2%)	2 (1.2%)	1.000
In-hospital/30-day mortality	2 (1.2%)	8 (4.7%)	0.054
Postoperative hospitalisation (days)	10 (9, 12)	12 (10, 15)	<0.01

Minor complication: Clavien–Dindo grade 1–2. Major complications: Clavien–Dindo grade 3–5

*RLN* recurrent laryngeal nerve

**Fig. 1:**
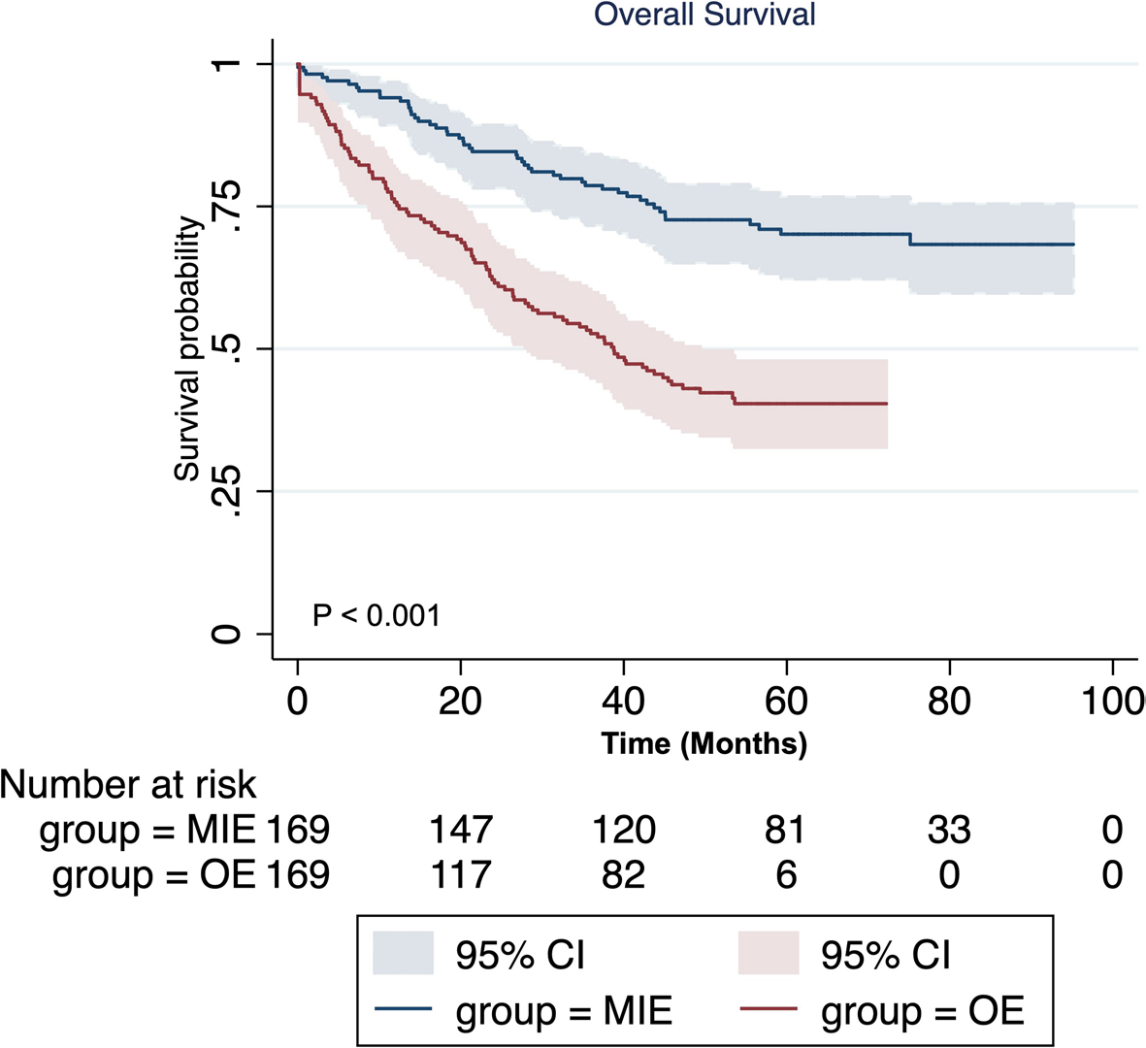
Comparison of overall survival between patients who underwent McKeown minimally invasive oesophagectomy and those who underwent open oesophagectomy

The range of follow-up was 15–74 months. As shown in Fig. 1, patients who underwent McKeown MIE had higher 1/3/5-year OS than those underwent OE (1-year: 94% vs 75.5%; 3-year: 78.5% vs 52.7%; 5-year: 69.9% vs 40.4%, all *p*<0.001).

## Discussion

In the current study, we found that McKeown MIE resulted in less bleeding, longer operative time, shorter postoperative hospitalisation, and showed a trend of lower in-hospital/30-day mortality than OE did. Although the rate of RLN injury was comparable between the two groups, lower incidences of postoperative pneumonia and anastomotic leakage have been observed in patients who underwent McKeown MIE. In addition, McKeown MIE was related to more harvested lymph nodes and improved survival compared with OE. The present study showed that McKeown MIE was safe and feasible for EC with similar or better surgical and oncological outcomes compared with OE.

Radical oesophagectomy is generally accepted as a standard surgical procedure for resectable EC. MIE was introduced to decrease the significant perioperative morbidity and mortality caused by OE [12, 32]. Several previous studies have demonstrated that MIE could significantly improve perioperative outcomes of EC; however, limitations existed in some of them, such as selection bias and data heterogeneity [22, 33–35]. The only level I evidence came from a recent multicentre randomised clinical trial [36–38]. These reports identified that, in comparison with OE, MIE was significantly related to less bleeding, lower pulmonary infection rate, and shorter hospitalisation. The present results were consistent with previous reports [12, 18, 30, 39, 40] that the blood loss was significantly decreased in the McKeown MIE group, even though the operation time was longer. Higher blood loss during oesophagectomy has been shown to be significantly related with the prognosis of EC [41]. Furthermore, McKeown MIE was associated with a shorter postoperative hospital stay than OE was and had a trend of lower in-hospital/30-day mortality than OE did. Meanwhile, the intra-operative morbidity and number of reoperations were comparable between these two groups. These results together suggest that McKeown MIE is a safe procedure.

The choice of incision and position are always the subject of extensive discussion for oesophagectomy. The cervical incision is necessary to create cervical oesophagogastric anastomosis and lymph node dissection during McKeown MIE. Since the chest incision in the McKeown MIE group was measured approximately 1 cm, the disadvantages of OE, for instance, extensive trauma, distraction of ribs, and damage of chest wall, which may lead to blood loss, can be avoided. Some surgeons choose to use Endo GIA staples to perform an intracorporeal gastric conduit [42, 43]. In our series, a GIA linear stapler was used to perform a small abdomen incision along the lesser curvature to prepare the gastric conduit extracorporeally. There are three advantages of this approach: (1) reduction of operating costs, time, and complexity, (2) easier removal of samples, and (3) more convenient indwelling of duodenal nutrition tube or jejunum colostomy.

The left-lateral semi-prone position of McKeown MIE could be another beneficial factor. Gravity and artificial pneumothorax can spontaneously expose mediastinal organs and structures that do not need any assistance. The lung then drops off regardless of small handling. Exudates aspiration is not needed during the surgery because the fluid mainly accumulates in the right-anterior chest cavity. In addition, the neutral position of the wrists and shoulder joints of the surgeon will minimise exhaustion and maximise ergonomic function. Meanwhile, intra-operative conversion to OE could be conducted without re-positioning patients and losing precious time as two conversions in our series.

Postoperative morbidity is one of the most concerning problems of oesophagectomy. McKeown MIE was related to a lower rate of postoperative morbidity in the current study, which was consistent with previous reports [44, 45]. Patients who underwent McKeown MIE experienced less pneumonia and anastomotic leakage compared with those who underwent OE. One of the most important differences between the surgical techniques was that a gastric tube was used for cervical anastomosis in McKeown MIE compared with the use of the entire stomach for the thoracic anastomosis in OE. Few studies have reported that cervical anastomosis was associated with a higher anastomotic leakage rate [46–49], given that available randomised evidence is limited. In contrast, Shen et al. [50] reported that a narrow gastric tube could reduce the incidence of MIE-related anastomotic leakage because of its relative longer tube of the stomach and less interference with perfusion. The present result is consistent with that of Shen’s result that less anastomotic leakage occurred following cervical anastomosis. It is noteworthy that all the patients in the MIE group received omentoplasty to cover the anastomosis which is also a possible reason to contribute to the current favourable results. A meta-analysis published on Cochrane Database Systematic Reviews [51] reported that the omentoplasty may decrease the incidence of postoperative anastomotic leakage for those who received transhiatal oesophagogastrectomy (RR: 0.23, 95% CI: 0.07–0.79); however, this benefit was not statistically significant on patients treated with transthoracic oesophagogastrectomy (RR: 0.19, 95% CI:0.03–1.03) or three-field oesophagectomy (RR:0.33, 95% CI:0.09–1.19). The role of omentoplasty on the reduction of anastomotic leakage after oesophagectomy warrants further randomised controlled trials. We also believe that it is associated with sufficient drainage, proper utilisation of antibiotics, and adequate nutritional support. Additionally, previous studies have shown that lower morbidity and mortality could be achieved because of the manageability of cervical leakage by cervical enterocutaneous fistula, compared with the thoracic anastomosis-related leakage [48]. Pleural and mediastinal infection caused by the anastomotic leakage is another risk factor for postoperative patients. In this study, three patients died of pleural mediastinal infection secondary to anastomotic leakage in the OE group.

Lymph node metastasis in the RLN region is a common progression of EC which results in poor prognosis [52]. However, both cervical lymphadenectomy and thoracoscope of McKeown MIE are believed to possibly increase the chance of RLN injury [53], resulting in the occurrence of complications and poorer survival [54, 55]. Hence, protecting the RLN during lymph node dissection is one of the key points, while carefully identifying and preventing accidental injury. The possible injury of heat conduction of ultrasonic scalpel is also worth the attention. Lymph nodes of the bilateral RLN were dissected as a routine for all patients, and the incidence of postoperative recurrent nerve injury was comparable between the two groups. The evidence above showed a combination of thoracoscopy and laparoscopy could provide a better vision field of surgery and the vascular and lymphatic vessels can be exposed more clearly.

Several studies reported that MIE was associated with a lower pulmonary complication rate compared with OE [13, 18, 36, 56–58]. In a recent study, Sihag et al. [13] reported that MIE was exclusively associated with a significant reduction in pulmonary complications. In contrast, Smithers et al. and other researchers [16, 59] suggested that MIE increased the incidence of postoperative pulmonary complications, indicating that the incidence of respiratory complications may vary based on the operators’ technique. In the present study, with regard to the incidence of respiratory complications, only the incidence of pneumonia (Clavien–Dindo grade 1–2) was significantly reduced in the McKeown MIE group compared with the OE group. The minimal lung retraction of McKeown MIE with less injury to lung parenchyma is thought to be one of the reasons that contribute to the reduction of pneumonia incidence. Furthermore, MIE reduced damage to the muscles of the chest wall, resulting in the easy drainage of bronchial secretions and less postoperative pain.

In addition to surgical technique modification, surgeons are also interested in improving oncological outcomes after surgery. The surgical margin affects prognosis after resection of the EC [60, 61]. It was initially suggested that MIE decreased the surgical margin due to the lack of palpation [41]. However, palpation probably is less important when a wide resection is planned, and it has been reported that the surgical margin and the rate of local recurrence were similar in the MIE and OE groups [62]. In the current study, all the enrolled patients underwent R0 resection.

Dissection of lymph nodes is another key factor for oesophagectomy due to its specific characteristics including multidirectional lymph flow and unpredictable lymphatic metastasis from cervix to abdomen [63–65]. Berger et al. found that MIE harvested more lymph nodes than OE did (20 vs 9) [66], which was consistent with the present results. Based on these results, the extended radical McKeown MIE with three-field lymph node dissection is confirmed to be a preferable surgical approach. Nevertheless, while studies suggested that MIE achieved equal or better oncological efficacy [38, 67], other studies showed no significant differences in oncologic efficacy between MIE and conventional OE [28].

Thus, the long-term survival was compared between the two procedures. In this regard, patients who underwent McKeown MIE had significantly longer overall survival compared with those who underwent OE. Palazzo and colleagues [68] analysed the 5-year survival of 168 patients with EC and demonstrated the superiority of MIE (hazard ratio 2.0). A previous meta-analysis showed a comparable prognosis among MIE and other surgical methods [69]. Another retrospective study, performed recently in UK, also reported significant improvement in survival by MIE compared with that by open or hybrid procedures [39].

Several limitations exist in the current study. One was the non-randomised design and limited sample size that needs further sufficiently powered RCTs. In this study, the PSM method was adopted to adjust for the confounding variables between the two groups. Moreover, all surgical procedures in the McKeown MIE group were performed by a surgeon with advanced thoracoscopic and laparoscopic experience and expertise. Thus, the reproducibility of this study may vary based on the proficiency and experience of the surgeons who performed the procedure.

## Conclusion

McKeown MIE is confirmed to be feasible and safe to achieve better surgical and oncological outcomes compared with OE. The McKeown MIE technology is superior to OE and is worthy of being widely applied in the treatment of EC.

## Data Availability

Please contact the corresponding author for data requests.

## Abbreviations

EC: Oesophageal cancer
OE: Open oesophagectomy
MIE: Minimally invasive oesophagectomy
RLN: Recurrent laryngeal nerve
ICU: Intensive care unit
